# “I’ve learned that I’m open-minded to this possibility”: A qualitative study to evaluate the acceptability of a psilocybin-aided smoking cessation treatment for people with HIV who smoke

**DOI:** 10.1186/s13722-025-00563-0

**Published:** 2025-07-21

**Authors:** Patricia A. Cioe, Garrett S. Stang, Danish Azam, Sarah Dugal

**Affiliations:** Center for Alcohol and Addiction Studies, Department of Behavioral & Social Sciences, Brown University School of Public Health, Box G-S121-5, Providence, RI 02912 USA

**Keywords:** HIV, Tobacco, Tobacco treatment, Psilocybin

## Abstract

**Background:**

People with HIV (PWH) are disproportionately affected by cigarette use, with a 40 − 70% prevalence rate. Although many express a strong interest in quitting, many PWH who smoke experience lower cessation rates with traditional treatments, in part due to their comorbid anxiety and depressive symptoms. Psilocybin, a classic psychedelic referred to as “breakthrough therapy” by the U.S. Food & Drug Administration (FDA), has been shown to have potential as a therapeutic treatment for psychiatric symptoms, (e.g., anxiety and depression) and substance use disorders, including tobacco dependence. Preliminary evidence has shown that administering psilocybin to people who smoke and have been previously unable to quit with traditional treatments resulted in impressive smoking abstinence rates (80%) at 6-months in a smoking cessation pilot study.

**Objective:**

Explore, using qualitative methods, the perceptions and acceptability of a psilocybin-assisted treatment for smoking cessation among PWH who smoke.

**Methods:**

Semi-structured, in-depth qualitative interviews were conducted with PWH who smoke. Interviews were audio-recorded, transcribed verbatim, and analyzed using rapid thematic analysis.

**Results:**

Twenty-five participants were enrolled: 15 cis male, 9 cis female, and 1 transgender female. Five main themes emerged: varying previous experiences with psilocybin; uncertainty about psilocybin’s effects and concern over potential side effects; need for trusted sources of information and testimonials; ultimately willing to try psilocybin-aided therapy for tobacco treatment; and, set and setting of psilocybin use matters.

**Conclusions:**

Psilocybin-assisted smoking cessation treatment appears to be acceptable among PWH who smoke. Participants highlighted the importance of addressing key concerns related to an emerging therapy to increase acceptability and willingness to try it. Further research is needed to evaluate the safety and effectiveness of psilocybin prior to incorporating this emerging therapy for smoking cessation into tobacco treatment clinical services for PWH.

**Supplementary Information:**

The online version contains supplementary material available at 10.1186/s13722-025-00563-0.

## Background

Cigarette smoking is more prevalent (40–60%) [1] in people with HIV (PWH), when compared with the general population, and is linked to increased morbidity and mortality [2]. Studies suggest that PWH who smoke (PWH-S) lose more years to smoking than to HIV infection itself [3]. PWH-S have increased rates of smoking-related illness, such as cardiovascular disease, pulmonary disease, and lung cancers [4, 5]. Cancer has emerged as a leading cause of morbidity and mortality in PWH [5–7], and a recent study demonstrated that the increased rate of lung cancers in PWH was attributable to cigarette smoking, rather than to immunodeficiency or HIV-related factors [7]. Further, it has been estimated that at least 90% of lung cancers and 20% of other cancers in PWH could be prevented by eliminating smoking [8]. Thus, the health risks related to smoking in PWH are substantial.

While most PWH-S report a strong desire to quit, they are less likely to quit when compared to the general population. PWH-S often respond poorly to traditional smoking cessation treatments, and smoking cessation rates are much lower among PWH. Smoking cessation trials among PWH to date have demonstrated disappointing outcomes, with low quit rates, poor adherence to cessation pharmacotherapy, and a lack of sustained abstinence [9]. PWH who smoke cigarettes are a particularly challenging group, often reporting high rates of comorbid anxiety and depression, factors that have been related to poor smoking cessation outcomes. Our qualitative work and that of others has demonstrated that PWH-S report that smoking helps them manage or alleviate the stress associated with living with HIV [10–12]. One study found that 80% of PWH-S reported that symptoms of anxiety and depression were barriers to maintaining abstinence following a quit attempt [13, 14]. Thus, designing approaches to address anxiety and depression in PWH-S may reduce symptoms, reduce potential barriers to cessation, and improve smoking cessation outcomes.

Generalized anxiety disorder and depression are the most commonly diagnosed mental illnesses among the general population in the US and among PWH [15]. Treatment is often in the form of selective serotonin reuptake inhibitors (oral medications), which are considered first-line treatment. However, many patients, including PWH, are reluctant to add additional medications to their often extensive treatment regimens [16], and instead prefer non-pharmacologic or alternative therapies [17]. Psilocybin, a classic psychedelic, has been shown to have potential as a therapeutic treatment for psychiatric symptoms, such as anxiety, depression, and substance use disorders, including tobacco dependence [18–22]. A psilocybin-assisted treatment option may be especially attractive to PWH-S.

Psilocybin-assisted treatment has been referred to as “breakthrough therapy” by the US Food & Drug Administration [23]. Recent clinical trials have demonstrated that 1–2 doses of psilocybin alleviated depressive symptoms with effects lasting as long as 12 months [19, 21, 24], and improved anxiety symptoms for at least 6 months [20]. Preliminary evidence has shown that administering psilocybin to people who smoke who have been previously unable to quit resulted in impressive smoking abstinence rates (80%) at 6-month follow-up in a smoking cessation pilot study [18, 22]. No funded projects to date have examined the effectiveness of psychedelic therapy for tobacco dependence in PWH-S. The overall goal of this research project was to examine the acceptability of psilocybin-assisted treatment among PWH who smoke cigarettes, and report that they are struggling with anxiety and depression as barriers to successful smoking cessation. Our specific aims were to: (1) explore, using qualitative methods, perceptions of psychedelic treatment: perceived benefits and harms, barriers, preferences, and likelihood of engaging in psilocybin-assisted treatment for smoking cessation; and (2) examine the acceptability of psilocybin-assisted treatment for smoking cessation among PWH-S.

## Methods

A comprehensive search of published articles examining the use of psychedelics in treating substance use disorders and mental illnesses such as depression and anxiety was conducted. While 21 articles (both qualitative and quantitative research reports) were selected for review, articles examining the effects of psilocybin in treating nicotine dependence and other substance use disorders were most relevant for the purposes of our study [18, 19, 22, 25–41]. Due to the role of depression in exacerbating nicotine dependence, articles relevant to the treatment of depression with psilocybin and other psychedelics were also included within the search. Based on our review of the literature, we developed a qualitative interview agenda (see Supplementary Files).

Participants were eligible for the study if they were at least 18 years old, diagnosed with HIV, smoked at least 5 cigarettes a day, and had a confirmed exhaled carbon monoxide (CO) level of 5ppm or greater as evidence of current cigarette smoking. Participants were ineligible if they were pregnant, expressed suicidal ideation, had uncontrolled high blood pressure, or had been hospitalized within the last month. Purposive sampling was used to recruit participants from an ongoing smoking cessation randomized clinical trial (RCT). Individuals who were enrolled in a 16-week smoking cessation randomized controlled trial for PWH were invited to participate in the qualitative interview at their week 12 or week 16 study visit. We also displayed flyers in infectious disease clinics and community-based organizations that provided care to PWH. Community partners were provided study flyers to distribute to patients if they were interested in participating in the research study. Eligibility screening was conducted by phone by research assistants (RAs) working within the Center for Alcohol and Addiction Studies at Brown University School of Public Health. Participant demographics, medical history, and smoking habits were extracted from the baseline data of the parent study, if they were recruited from the ongoing study, or by self-report if they had responded to clinic and community flyers. All participants were compensated for their participation.

Brief, semi-structured qualitative interviews were conducted, via Zoom or in-person, between June 2023 and April 2024. Participants completed the informed consent process prior to completing the interview. After providing a brief introduction about the purpose and format of the interview, the Principal Investigator (PI), a master’s-level RA, and a masters’ student RA, all experienced in qualitative research methods, conducted one-on-one face-to-face interviews in a private room following an interview guide. The RAs collected demographic data, medical history, and smoking history via a Qualtrics survey from each participant. For those participants who were concurrently enrolled in the RCT, this information was extracted from the parent study data. Four open-ended questions were asked to gather information on the overall interest of using psilocybin for the purpose of smoking cessation and to assess the acceptability of participating in a pilot study for psilocybin-assisted smoking cessation treatment among PWH. The interview, which lasted approximately 30–40 min was divided into three main sections: (1) general views on psychedelics, including past experiences and their medical applications, with a specific focus on psilocybin for treating nicotine dependence and other mental health conditions; (2) perceptions of health, covering the perceived benefits and risks of using psychedelics; and (3), participants’ willingness to consider psilocybin-assisted treatment for smoking cessation. Interviews were audio-recorded and transcribed verbatim. Audio recordings from the in-person and Zoom interviews were stored in a secure drive at Brown University and were sent for professional transcription. All participants were assigned a study ID; names were redacted within the transcriptions to ensure confidentiality.

### Ethical considerations

The Brown University Human Research Protection Program/Institutional Review Board (IRB) approved this study (STUDY00000064) on June 28, 2023. All participants provided written informed consent.

### Data analysis

#### Quantitative analysis

All analyses were performed using RStudio [42]. Descriptive statistics were calculated for all demographic, clinical, and smoking history variables.

### Qualitative analysis

The transcripts were reviewed and checked for accuracy with the audio-recording by the RAs. The qualitative data analysis software Taguette, a free, open-source computer-assisted qualitative data analysis software package developed for qualitative research thematic analysis [43], was used for coding the transcripts. An initial coding structure was developed from the semi-structured agenda and was refined as the coding process continued to include emergent themes. Each transcript was individually coded by the RA who conducted the interview and then double-coded by the PI. Using an iterative analysis process, the RAs and PI met weekly to discuss and resolve coding discrepancies and to identify points of consensus. Codes were summarized and five key themes were identified; illustrative quotes were selected to reflect each theme.

## Results

Thirty-three potential participants were screened: 25 participants were eligible and enrolled; seven were eligible and scheduled for the appointment but did not show up for the interview; one was ineligible as they were taking smoking cessation pharmacotherapy and had quit smoking at the time of screening. Twelve participants had participated in the concurrent smoking cessation trial, while 13 had not. Eight interviews were conducted in person and 17 were conducted via Zoom. Table 1 summarizes the sample demographics and characteristics.

**Table 1:** Participant demographics, characteristics, and smoking history (*N* = 25)

	Overall Mean (SD) or *N* (%)
Age, in years	56.40 (12.48)
Sex at Birth	
Male	16 (64.0)
Female	9 (36.0)
Gender Identity	
Cis Male	15 (60.0)
Cis Female	9 (36.0)
Transgender Female	1 (4.0)
Race	
White	16 (64.0)
Black or African American	5 (20.0)
American Indian or Alaskan Native	2 (8.0)
More than One Race	2 (8.0)
Ethnicity	
Hispanic	3 (12.0)
Education	12.96 (2.95)
Employment Status	
Employed full-time	4 (16.0)
Employed part-time	2 (8.0)
Retired	8 (32.0)
Disabled	7 (28.0)
Unemployed	3 (12.0)
Self-employed	1 (4.0)
Years Living with HIV	23.12 (11.97)
Diagnosed Mental Health Disorder^a^	17 (94.4)
Number of Lifetime Quit Attempts	5.80 (6.06)
Cigarettes per day	11.64 (6.40)
Years Smoking Cigarettes	36.24 (15.97)
Cigarette Dependence (FTCD)	4.00 (2.10)
Confidence to Quit (scale 1-10)	6.64 (2.38)
Previously used Tobacco Cessation Product^b^	20 (80)
Previously used Psilocybin	14 (56.0)
Previously used Psychedelics^c^	16 (64.0)
Willingness to engage in Psilocybin-aided therapy^d^	24 (96.0)

FTCD = Fagerstrom Test for Cigarette Dependence

^a^ Mental health disorder could include depression, bipolar disorder, panic disorder, obsessive-compulsive disorder, PTSD, psychosis, or anxiety disorder

^b^ Tobacco treatment could include nicotine patch, gum, lozenge, inhaler, nasal spray, or e-cigarette, bupropion, varenicline, or behavioral (talk) therapy

^c^ Psychedelics could include LSD, Mescaline, Psilocybin, MDMA, etc

^d^ Psilocybin-aided therapy could include any form of medical therapy that involves Psilocybin, not just tobacco

The interviews generated five themes that characterize key factors describing perceptions, acceptability, and willingness to engage in psilocybin-aided therapy for tobacco treatment: (1) varying previous experiences with psilocybin, (2) uncertainty about psilocybin’s effects and concern over potential side effects, (3) need for trusted sources of information and testimonials, (4) willingness to try psilocybin-aided therapy for medical and/or tobacco treatment, and (5) set and setting matters. Each illustrative quote is annotated with the participants’ age, gender identity, and whether they had previously used psilocybin; additional quotes supporting the five themes are presented in Table 2.

**Table 2:** Qualitative themes and additional illustrative quotes

Theme	Illustrative Quotations
**Theme 1. Varying previous experiences with psilocybin**	What made it enjoyable? The—the mental exploration. Oh. Free-thinking. – 502, cis male, 60 years old, previously used psilocybin it was—it was really—it was nice, and I really didn’t—I didn’t—I thought I’d get sick I felt, like, really good, happy. Um, let’s see, I definitely wasn’t depressed. I was like more, like—if it, like—I’ve only taken it at, like, concerts, and it gets you into the concert more, and you—you enjoy yourself. You’re happy; you laugh—but I’ve never taken ‘em, like, by myself, I took the s—the mushrooms, like, there was no depression. I was so happy, and joyful, talkative. So we could get it—we could get along with each other, like, and have—have a good time, party, and whatever, and—and you can walk for miles, and miles, and miles, and miles, and it doesn’t affect you. And you get a crowd of people together, and you’re all taking it. You’re all getting along. You’re all partyin’, laughin’, smilin’. – 515, cis male, 53 years old, previously used psilocybin No. I used to do it in the midst of a crowd. And then enjoy the—the surroundings. Uh, everything seemed to be amplified, you know. And it—it—it tended to make me feel so much more happy. Yeah. You know? – 516, cis male, 70 years old, previously used psilocybin I prefer psilocybin to LSD, because it’s not as intense, I—I love the psilocybin because of—it’s a gradual, you know, gentle, not just punch you in the face and there you go. Um, i-i-it’s just, um, the psilocybin is a more enjoyable psychedelic experience because it’s—a gradual. It’s a crescen—it cre—it’s got a crescendo. And then it’s got a decrescendo. It’s not just the “Hello. Here you are. The opera’s all over all at once.” And, you know, you’re left wanting more, and, yeah, you know, i-i-i-i- it’s just—it’s more gradual. It’s easier. It’s better tolerated. – 520, transgender female, 40 years old, previously used psilocybin
**Theme 2: Uncertainty about psilocybin’s effects and concern over potential side effects**	…you can have that bad trip and think somethin’ is somethin’ and then you just freak out and then kill somebody yourself, you know?…Because you can bring back all them memories too. You know? Because you might sa—you can think you was back in Vietnam if you’re on a—if you’re on that kind of a—a—a trip with that. Someone can think, “Oh, he’s in the bushes!” I mean, people think that without being on stuff, and they give them some of the medicine they give them now is to stop that, and it’s not stopping that…They still think there’s people coming out of the bushes, gonna grab ‘em and stab ‘em and shoot ‘em and stuff. – 512, cis male, 58 years old, previously used psilocybin Um, if you take ‘em by yourself, and you have a bad tri—you could, like, do some stupid stuff—either killing yourself or killing somebody—and you get paranoid. I know you can get paranoid on it. Um, and i—you do stupid stuff, like, you could think, like, you might—you might be trippin’, and you think you can fly. So you decide, “Oh, I’m gonna go up on that building. I’ll show you I can fly.” And, uh, you don’t fly: you—you hit that concrete. You’re done. – 515, cis male, 53 years old, previously used psilocybin Um, that I could have a bad trip and not come back. ‘Cause some people get stuck in a bad trip from using psychedelics. – 523, cis male, 42 years old, previously used psilocybin [I’d like] the first time to be in a controlled environment. I don’t wanna be tripping riding my bike. Like, if I’m tripping and I don’t—like, don’t know where I am. And I like, if I lose all sense of anything I can’t—there’s no self-protection. There’s no looking out for myself or anybody else around me… As long as I’m not using too much, then I’m fine. I don’t like being out of control. – 502, cis male, 60 years old, previously used psilocybin There’s some people having a bad reaction and having a bad trip. That—that would be my real biggest concern…. People having adverse reactions to it. – 520, transgender female, 40 years old, previously used psilocybin I was always afraid of them. I was afraid of those. I—I heard people went crazy on it. That’s why I was afraid of it… I don’t wanna put anything—alter anything in my body ‘cause of my health. I—I have to stay on a straight and narrow path with my health. Especially with my virus. I take so many medications now I—I don’t wanna add to it if—unless I have to. I—I—it’s—it’s a combination of the medicines with that. It—I don’t know what the effects would be to my body. I’m trying to keep a fresh brain every day ‘cause I got health issues. Um, for me? Um, it could m-make something else in my body worse than it is. Like—like, um—or I could get sick and not get better from it. I don’t know what the side effects of it would be. Basically that’s what I’m saying. You know? – 510, cis male, 57 years old, never used psilocybin Um, [I’m] afraid that it’s gonna do something to me. Side effects. Um, the stomach. Sometimes side effects affects your, um, kidneys. Sometimes they affect your liver. Maybe your heart too…I have no idea what causes you to have some side effects that messes with your liver and kidneys. – 514, cis female, 69 years old, never used psilocybin Um, second, is, um, I—I guess safety is the most important, um, thing that I have in mind. You know? Um, and, um, um, other than that, I don’t really have any other concerns. It’s really about safety an-an-and, you know, um, and—and bein’ mon—and bein’—bein’ monitored. – 517, cis male, 67 years old, previously used psilocybin I don’t—I would—I don’t know how it’s gonna affect me. – 518, cis female, 64 years old, never used psilocybin Because I have an addictive personality, my concern would be that I would abuse—I would abuse it. – 507, cis male, 70 years old, never used psilocybin You know, because, you know, we’re—we’re dealing with something i—that—that i—could lead to another addiction…the risks, um—you know, I don’t know that I—it—I didn’t—I didn’t experience anything that would go to what I would call an addiction let’s just say that I—I think it’s, er, the most important thing is that it be very, very controlled, because—mainly because of, er, I guess…um, my fear of—it—it—I—although I don’t know anyone who—who up—who got to a point of, uh, addiction. But I think the possibility exists… because of the fear of addiction or—or—and because we’re dealing with something that is… it’s—it’s nontraditional, but it is psychedelic. – 521, cis male, 70 years old, previously used psilocybin
**Theme 3: Need for trusted sources of information and testimonials**	I think there was one that I saw on Netflix that was pretty exhaustive about—ab- about usage—um, not specifically for smoking sensation [cessation]. It—it was for, you know, various other things. – 521, cis male, 70 years old, previously used psilocybin Now, when I go home, I’ll Google and I’ll start looking. I will. That’s how I am. I’ll be home, and then I’m Googling, looking up mushrooms and reading about it. – 504, cis female, 60 years old, never used psilocybin I just word of mouth from professionals that—that said that they had some really g-good outcomes with—with, you know, using, uh, uh, psychedelics. Well—well they’re doin’ studies now so, and from what—from what I heard, they’re—y—y—w—they have a very—good outcome, um, on the—on—positive outcomes with, um, some—with some veterans who did suffer from anxi-anxiety, some, um, some—some mental health issues, um, such as depression. Um, also addiction, um, things like that, so they’ve—they’ve had some really good positive, uh, outcomes. – 517, cis male, 60 years old, previously used psilocybin Like I said, I’ve had friends who have done the research aspect of it for depression and they found it very useful. They found it very useful, and they’re now using it. – 502, cis male, 60 years old, previously used psilocybin
**Theme 4: Willingness to try psilocybin-aided therapy for medical and/or tobacco treatment**	I would definitely be interested to find out, like, how it would help with, like, my depression and smoking definitely. – 515, cis male, 53 years old, previously used psilocybin Um, quitting smoking? I would definitely say, “Try it,” because it does have, you know, the effects of reducing the cravings as well as, you know, the antidepressant properties as well as mood stabilization properties, um—I mean, it’s attractive to me as is, however, it—the—the—you know, how—knowing that there could be extra benefits to it, yeah. Because a lot of peop—uh, people who do smoke cigarettes also have comorbid, uh, mental illness, like depression, anxiety. – 520, transgender female, 40 years old, previously used psilocybin
**Theme 5: Set and Setting Matters**	Hmm, a-a-arts and crafts. Things that—something that’ll keep them stimulated. Something that can—they can put that new perception into. – 516, cis male, 70 years old, previously used psilocybin Something comfortable. You know, like maybe somebody could lounge, relax. You know. TV, I don’t know just to make you feel comfortable. Maybe bring a friend with you. – 524, cis female, 38 years old, never used psilocybin Actually, I think there’s a benefit to a group in that afterwards, people could share their experiences so that you can find if there’s a synergy, uh, amongst everyone. So, I think that’s very beneficial. – 507, cis male, 70 years old, never used psilocybin

### Theme 1: Varying previous experiences with psilocybin

Participants with previous positive experiences often highlighted that psilocybin was enjoyable and improved or stabilized their overall mood. These individuals frequently endorsed significant increases in feelings of happiness, reduced depression, heightened sense of self and surroundings, and amplified social ability.

*It was like hallucinogenic, and you’re kind of happy, and like, you know, it was like—I can’t say it was—uh, in a way I can say it was heavenly, you know?* – 511, cis female, 66 years old, previously used psilocybin.

*Well, it helped me, as far as mental health, because I would—once I found out I have HIV in 1986, people just c-co-completely threw me off, and was—said “oh, no, no,” they were all s—afraid of everything…. It gave me strength. I was able to go out and do my stuff, and continue on. And then it also became kind of a medicine. It—and it helped me relax on myself, and not be worried about what’s happening outside of me, with all the difference between means and people [? ]. It helped my mental health. Stay strong, stay positive, and keep going. ’Cause whatever it is, just stay with it. It ain’t about what other people say, and this and that. Like, it was, like, no. ’Cause life was—was going to be what it was going to be, which is what the mushrooms taught me. So I—have learned to become p—stay positive. And t—and then to this day, I’m still positive, I feel, because of the—the usage of these certain things during my life.* – 516, cis male, 70 years old, previously used psilocybin.

Adverse experiences were characterized by psychological responses, including dissociation, vivid hallucinations (plus dermatillomania), and paranoia. Some individuals noted having a taste aversion to ingesting psilocybin-containing mushrooms (often referred to as “magic mushrooms” or “shrooms”).

*Really can’t think of that much. I didn’t know. ‘Cause I tried ‘em one time, and it was like, no, nope. And then gone on. Didn’t do it again. And it tasted like nasty—moldy stuff. And, it’s not like, I guess.*

*the mushrooms they sell today. I mean, maybe they’re flavors; I don’t know. But this was just, white mold. I did not like it. I would—I never—I tried it one time and never again did I.* – 503, cis female, 59 years old, previously used psilocybin.

*Um, I just didn’t enjoy being on that kind of high. Um, I think it was just too intense, too—too high. slightly paranoid. Um, anxious.* – 505, cis male, 67 years old, previously used psilocybin.

Participants who reported neutral experiences noted that psilocybin had minimal effects and did not produce noticeable experiences. It is worth noting that some participants noted both positive and neutral past experiences involving the use of psilocybin, indicating variations in past “highs” or “trips.”

*Yeah, I tried, um—I tried twice. I tried, uh—during the pandemic, uh, my business partner brought some for me to try that he grew at home, and—but really I felt most-mostly nothing. I didn’t feel dizzy. Didn’t have any illusion. Didn’t feel anything. It was like I really had nothing. And the second time was in Brazil, and, uh, I was just—just chilling in the sofa, watching Netflix movie all afternoon. It was a rainy day. We didn’t do much at all. So I really don’t know how much that affected me either. I don’t really feel like I had any kind of experience.* – 509, cis male, 52 years old, previously used psilocybin.

*I don’t look at it as a negative, um, you know. But is it something to chalk up as—as being ver—a—a positive influence? I can’t say that it had that much of effect.*– 521, cis male, 70 years old, previously used psilocybin.

Eleven participants had no prior experience with psilocybin, either due to lack of interest, awareness, or reports of negative experiences from others who had tried psilocybin.

*I don’t know nobody that ever used it.*– 508, cis female, 63 years old, never used psilocybin.

*I’ve n—I never. I was always afraid of them.*– 510, cis male, 57 years old, never used psilocybin.

*I don’t really know—this is the first time I heard of psychedelic mushrooms and stuff like that. So I, you know—I don’t know how to comment on it. You know what I’m saying?*– 518, cis female, 64 years old, never used psilocybin.

### Theme 2: Uncertainty about psilocybin’s effects and concern over potential side effects

There was a general consensus expressed by all study participants about the fear of the unknown related to psilocybin-assisted therapy.

*Um, I don’t know. I’d probably have to know a little bit more about it… like is it par—like, some drugs can make you paranoid. I need to know a little bit more about it…*– 504, cis female, 60 years old, never used psilocybin.

*As long as I don’t start seeing things… I don’t wanna f-feel as though like I’m on some cloud nine, or like if I was like seeing all purple or a elephant like walking by, and I don’t wanna seen none of that. That’s what I would be afraid of.*– 508, cis female, 63 years old, never used psilocybin.

Some participants expressed apprehension about loss of control during the treatment experience, uncertain about how psilocybin would affect them. Many of these concerns were based on experiences reported by others who had previously used psychedelics recreationally.

*I just don’t like to lose control about anything. Um, so anything that could possibly feel like I don’t have control about my body, my s—the—the—the environment when I’m in, uh, I don’t th—I don’t think it’s gonna be something that I—I—I will keep using.*– 513, cis male, 38 years old, never used psilocybin.

*‘Cause when—when you tr—when you—when you eat mushrooms, it makes you think a lot.*

*It makes my brain, like, go crazy and, uh, think a lot of stupid things, you know. Um, I don’t know, like, you have to be prepared for, um, laughing and, like, um, I don’t know. The—the things that you have close to you, like, it has—it has to be, like, safe.*– 525, cis male, 31 years old, previously used psilocybin.

Participants also expressed concerns about interactions with their antiretroviral therapy, their HIV virus, and potential impact on their overall health.

*Uh, well, just put—put me at ease with any interactions with other drugs that I might be taking, so I’m not worrying about that kind of thing. If you take that out of the equation, I’m not worrying about that. That’s—that’s kind of—yeah, that’s basically it. Interaction with other drugs. You know, giving me some kind of seizure or something.*– 509, cis male, 52 years old, previously used psilocybin.

*I’m scared to do stuff like that. And I already have enough health issues going on that I don’t want anything else to… you know mess me up. Maybe I would hallucinate too much, and it would make me go crazy or something, or make me scared. And yeah, it’s just I don’t know. It’s just something I just don’t wanna try. Does it like mess with your heart, or like your blood pressure? Things like that. Yeah, and that’s why I don’t really know, if I would want to do that.*– 524, cis female, 38 years old, never used psilocybin.

While many participants were worried about the potential side effects of the treatment, others expressed concerns about addiction. Some participants feared that they might enjoy the effects of psilocybin and seek to use psychedelics beyond the treatment session.

*Well, I mean, they’re gonna be addictive. Once the study’s over, you know, I don’t wanna start looking for ‘shrooms. Well that’s a fear. I mean, I don’t want to be—I’m addicted to one drug now [tobacco], and I don’t want to be addicted to another.*– 501, cis male, 68 years old, previously used psilocybin.

*I might like it. ‘Cause if—if me—if I took it and I enjoyed it, then I’m gonna always want it to feel that way, and that’s something I don’t wanna do. I’m afraid of—the only addiction I ever had was cigarettes—and I’m afraid of addictions. Well, I protect myself because I don’t be around it; I don’t be around it. And like this mushroom thing you’re talking about, “Supposing I like it?” That’s the fear—and just like any addict—they tried it one time, and they ended up liking it—I don’t wanna like it and I want it all the time—because I can’t say, “Oh, I would never,” because you never know—so that’s what I’m afraid for.*– 508, cis female, 63 years old, never used psilocybin.

### Theme 3: Need for trusted sources of information and testimonials

Participants expressed a desire for a greater understanding of the therapeutic applications of psychedelics across different medical conditions and behaviors before contemplating their use.

*I’d rather see more research…. I would want to see more, results in the future before I would do something like that.*– 524, cis female, 38 years old, never used psilocybin.

*I would have to do more research. Right. I wouldn’t just say, “Okay, I’ll do it,” I’d wanna read more about it— I’d wanna Google more about it—at least get—find more information about it. That’s what I’d do.*– 508, cis female, 63 years old, never used psilocybin.

Some emphasized the importance of clinical trial outcomes for reassurance, while others mentioned relying on online sources.

*Well, I’ve been—I’ve been seeing articles, uh—scholarly articles, not just, you know, not the Rolling Stone magazine articles, but you know, something that—that has been peer reviewed. And I’m—I’m seeing that there’s a lot of benefit that is suspected. I know… they’re starting to use them in, um, psychiatry.*– 507, cis male, 70 years old, never used psilocybin.

*you know, like… it’s gonna take—it’s going to take trials to—to see how it, er, you know, er, if it—if it does produce the results—the desired results. I was—I was always willing to—to participate, because let’s face it, you don’t—you don’t find out what works until you get through these trials. I’m always in support of, um… of trials.*– 521, cis male, 70 years old, previously used psilocybin.

Encouragement and advise from healthcare providers were cited as influential for those considering psychedelic treatment for smoking cessation.

*I can talk to my doctors about getting on it, you know, and tell them that I’ve had the experience with it before, and I do have somebody who is, you know, in some kind of behavioral health, slash, harm reduction, slash, you know, er, er, research studies to back me up, you know, with hearing my results and hearing the results of others.*– 520, transgender female, 40 years old, previously used psilocybin.

*If my doctor said it was good, I was okay, I would do it. If he approved it, ‘cause like I said, I would have to investigate and have to s—talk with my doctor in—just in case there’s a bad side effect. I would know what’s wrong with me. But—but, um, like I said, I—I would give it a ch—if—if Dr. < Name > said, “< Name>, give it a shot,” I would give it a shot. Anything that could help me quit smoking.*– 510, cis male, 57 years old, never used psilocybin.

Additionally, hearing positive feedback from trusted individuals who had personally benefited from such treatments was highlighted as a motivating factor to consider trying psychedelics.

*I just—I, uh, I-I’m not like this. Um, I just—of course, uh, uh, hearing the testimony b—from—from other people—for whatever reason, ob-obvious, it’s gonna be helpful.*– 513, cis male, 38 years old, never used psilocybin.

*I would also like to—someone—then to find out someone used it first, and how they experienced it. If I had positive—um, people say positive things about it ‘cause one person has to tell me that they went on some kind of trip I would never take it, but if people came to me and said, “That’s something,” then I would try it.*– 508, cis female, 63 years old, never used psilocybin.

*I’m gonna listen to some—a counselor who did drugs, not somebody who never did drugs. I’m gonna listen to the person what they experienced, not from somebody who just read a book, not someone who didn’t, and just says, “Oh, well, it’s gonna work.” No, you show me that, “< Name>, listen, I took this, and it’s, um, it’s helping me with my depression, um, I’m feeling this type of way, I’m not on no cloud,” you know, “I’m relaxed,” you know, and “I stopped smoking, I’m not physically stopped, but I’m on the verge of stopping,” that’s something to think about, you know? I would listen to that person— and because they experienced it. Um, like I said, if someone did the program, and still in the program, and s—you know, I would love to do it—I would love to, but I would definitely have to find out some more information on it*.– 508, cis female, 63 years old, never used psilocybin.

### Theme 4: Willingness to try psilocybin-aided therapy for medical and/or tobacco treatment

Most participants (*n* = 24, 96%) were open to psilocybin-assisted therapy and expressed willingness to consider the treatment for medical and tobacco-related purposes. They often mentioned previous positive experiences, improvements to mental health (including alleviation of depression and PTSD symptoms), and a strong desire to quit smoking as motivating factors.

*Because I’m familiar with what taking a lot of it does—so microdose under—like—like—in a controlled study, absolutely it would be fine. Just, I don’t know, I have confidence in you guys.*– 502, cis male, 60 years old, previously used psilocybin.

*Yeah, definitely, it seems like a good thing, like you’re k—you’re killing, like, 2 birds with one stone.*– 514, cis female, 69 years old, never used psilocybin.

Several participants mentioned that they had tried and been unsuccessful with all other forms of tobacco treatment and were, therefore, willing to “try anything” to quit smoking.

*I think I’ve done them all [smoking cessation treatment]. Um—hypnosis, acupuncture, uh, patches, gum, Wellbutrin, um, uh, changing brands, uh, uh, you know, cutting back. A-and none of them have worked so far. So— I’d be willing to try anything.*– 507, cis male, 70 years old, never used psilocybin.

*I’ve learned that I’m open-minded to this possibility.*– 505, cis male, 67 years old, previously used psilocybin.

Some participants noted that they did not have concerns with this form of psychedelic compared to others and compared it to the use of marijuana, which is legalized for recreational use in the state where the study was conducted. While some participants did note hesitation initially, they ultimately endorsed their willingness to engage in future treatment.

*Um, I—it’s a particular problem down here. Uh, I look at psychedelics on a different level than I would, uh, crystal meth or heroin or, you know, whatever, uh, the real illegal—the hardcore, very addictive, illegal drugs. I view mushrooms, in my ignorance, on the same scale as I now consider marijuana. Well, I—I consider that, uh, there are applications for it, which are perfectly fine. And it’s—it should be—I don’t think marijuana is addictive. And I doubt that mushrooms would be addictive. It’s the personality. If they’re trying—if they’re looking for an escape, I don’t think they’re gonna find it in mushrooms ’cause they’re not going to find it in marijuana. So, I don’t wanna use the word harmless, but something that is less—less harmful, uh, still long-term. But I’d have to think about it.*– 507, cis male, 70 years old, never used psilocybin.

Participants who were initially hesitant to use psilocybin-assisted therapy expressed concerns about its general effectiveness and potential side effects, as well as skepticism regarding its efficacy in aiding smoking cessation. Only one participant (524) endorsed that they were completely unwilling to engage in psilocybin-assisted therapy because of concerns about the potential interactions with ongoing health conditions and potential side effects.

*I—I wouldn’t mess with it. Me personally. I—I just don’t feel it’s no—something I would want.*

*People was getting sick from it, and I don’t know if they got it from a study group, and—or off the street. So—I really don’t know. I don’t even like mushrooms. I’ve seen people get really messed up with mushroom, and I don’t know what type of mushroom it was or anything.*– 519, cis female, 56 years old, never used psilocybin.

*I—I—I wouldn’t use it for that [tobacco tx]. No.*– 512, cis male, 58 years old, previously used psilocybin.

*I’m scared to do stuff like that. And I already have enough health issues going on that I don’t want anything else to… you know mess me up. Maybe I would hallucinate too much, and it would make me go crazy or something, or make me scared. And yeah, it’s just I don’t know. It’s just something I just don’t wanna try.*– 524, cis female, 38 years old, never used psilocybin.

Some expressed concerns about the potential for psilocybin to interact with their HIV virus or other comorbidities. They expressed a need for more information and additional research evidence before confidently trying this treatment.

*I wouldn’t even—I wouldn’t s-study—I wouldn’t even attempt to do—I don’t wanna—I don’t wanna put anything—alter anything in my body ‘cause of my health. I—I have to stay on a straight and narrow path with my health. Especially with my virus. You know, like I said, I got the HIV, I got third stage kidney disease, I got congestive heart failure, um, I—I got osteosporosis all in my bones. I was told I have the bones of a 85-year-old man. And I just don’t wanna—I don’t wanna go against, uh— with something that I don’t know about.*– 510, cis male, 57 years old, never used psilocybin.

*Um, I think they’d have to do a little bit more, like, uh, research in it, like, because it definitely wou— I—I don’t think it would be tough to get—you’d have to do a lot, a lot, a lot of research, probably, for, like—at least a couple years, I be—I imagine before they can go to the FDA—the FDA, and say, “Hey, look, this works for PTSD depression, smoking, and—and these are the side effects. These are the pr— the—the—these are the positives, and these are the negatives of it. But there’s more positive than they’re negative.” But yeah, you’d definitely, like, before it would get approved by the FDA, I think you’d have to have a lot of research on it.*– 515, cis male, 53 years old, previously used psilocybin.

### Theme 5: Set and setting matters

Most participants believed that a positive setting, which fostered a calm mood, would significantly enhance the psychedelic experience and lead to better health outcomes. They emphasized that comfort would be crucial for reducing the risk of a bad experience and mitigating potential negative side effects associated with psychedelic use. A well-designed, comfortable setting would help prevent adverse effects and would support a serene environment during the experience.

*Just some place where I can be relaxed, just enjoy it. Not have to move around, or go outside, or anything like that.*– 501, cis male, 68 years old, previously used psilocybin.

*’Cause music is—is universal as far as I’m concerned. It helps in many situations. ‘Cause, I mean, you really even e-e-estimate a person by what music they listen to. You know, how—how much of a—of a radical they are. Or how—how inno-innovative they are. Yeah.*– 516, cis male, 70 years old, previously used psilocybin.

*I would honestly say, some kind of relaxing environment. Like a—like a tre—like a spa treatment kind of thing. Like, incense, you know. Tranquil music. You know, uh, waterfall. Some kind of setting where it’s peaceful and you can feel Zen.*– 520, transgender female, 40 years old, previously used psilocybin.

Additionally, having a healthcare professional present was considered vital not only for optimizing health outcomes but also for ensuring participant safety and security.

*I’d rather be around some professionals that know what they’re doing and what’s—what’s—what to look for and what not to look for—versus me going home trying to figure it out myself and then do something foolish.*– 506, cis male, 60 years old, never used psilocybin.

*As long as I’m being monitored, I’m fine with that.*– 519, cis female, 56 years old, never used psilocybin.

## Discussion

This formative study explored perceptions of psilocybin-assisted therapy for smoking cessation among PWH who smoke cigarettes and found that, overall, participants were open to trying psilocybin as an alternative treatment. However, they had specific concerns that would need to be addressed in a future study’s design and procedures. The findings suggest that specific conditions must be met to ensure successful recruitment and enrollment in a clinical trial of this nature. This study also found that many PWH had previously used psilocybin or other classic psychedelics. They were hopeful about having a positive experience but had concerns about potential side effects or interactions with their antiretroviral medications. They expressed a desire for more information and testimonials from trusted sources and had specific preferences regarding the setting for dosing sessions.

More than half of those interviewed had tried psilocybin and other psychedelics in their lifetime and 24 (96%) indicated a willingness to try it for the purposes of smoking cessation. Overall, past experiences with psilocybin were positive with participants describing the effects as peaceful and “mind expanding.” This is encouraging as a meta-synthesis by Crowe et al. (2023) identified three key themes regarding the use of psilocybin among individuals with chronic health conditions, including HIV [28]. Participants reported that psilocybin facilitated new perspectives on their conditions, encouraging acceptance and motivation for change. It fostered a sense of connectedness to the world and an awareness of how their actions affected others. Finally, participants experienced a sense of transformation, releasing them from past constraints, enhancing self-appreciation, and leading to a renewed sense of identity. These effects could be particularly beneficial for individuals who have long identified as people who smoke, as they may facilitate a successful quit attempt.

Participants indicated a clear preference for a set and setting that would promote relaxation and comfort. They believed that a calm, relaxing, comfortable environment would not only be preferred but also might contribute to a more positive psilocybin treatment session. Creating a space that was calm and inviting could potentially improve the participant experience and influence the outcomes of the intervention. They emphasized that music would enhance the experience as well. Finally, adding stress-reducing elements such as aromatherapy or gentle background sounds could be appropriate and well-received by participants. Strickland and colleagues (2020) discussed the use of music during psychedelic therapy and pointed out that music is not only a standard feature of this type of therapy but also mechanistically relates in neurobiological and neurochemical ways to psilocybin [44]. They compared Western classical music with overtone-based music and participant-selected playlists in a sample of 10 participants enrolled in a psilocybin-assisted treatment session for smoking cessation. Despite the historical preference for classical music in such sessions, the study found no significant differences in outcomes between the different music types. Their findings suggested that developing a process for generating patient-specific musical selections rather than providing standardized music may improve therapeutic outcomes. Much consideration would be essential when designing a space for participant sessions in this type of trial. Implementing these aspects thoughtfully could not only enhance participant satisfaction but also contribute to the overall effectiveness of the intervention.

Participants indicated a strong desire for more information about psilocybin from trusted sources and noted that a recommendation from a trusted healthcare provider would be particularly impactful. Participants also reported increased willingness to undergo psilocybin treatment with a provider or a scientist (researcher) that was trusted due to their expertise and perhaps past experiences with the provider or scientist. This aligns with social cognitive theory, which highlights the importance of verbal persuasion, and the theory of triadic influence, which underscores how social contacts can affect an individual’s behavioral decisions. Gaining the support of and collaborating with HIV medical providers to facilitate enrollment in a psilocybin-assisted treatment for smoking cessation would be beneficial.

To our knowledge, this is the first study to explore perceptions and acceptability of psilocybin-assisted therapy for smoking cessation in PWH who smoke cigarettes. It does, however, have some limitations. The sample was small (although consistent with qualitative research sample sizes) and from one geographic area in the northeast United States. Hennick and colleagues, have noted that generally 9–17 individual interviews are sufficient to achieve saturation in qualitative studies [45]. Nevertheless, the findings from this study may not be generalizable to all PWH-S. Secondly, there may be some sampling and recruitment bias as half of the participants in this study were interviewed after participating in a tobacco treatment clinical trial for PWH-S. The findings may be different from people who smoke and are not interested in quitting smoking. Participants’ motivation to quit smoking in this study was high and this might have affected their responses, as indicated by the mean confidence in quitting. Finally, the majority of the sample was above 50 years of age and may have been familiar with or exposed to psychedelic use in the US during the 1960s, and may have used psychedelics themselves, possibly making them less apprehensive about trying a psilocybin-assisted treatment.

In sum, this formative research has several implications for future work. PWH who smoke cigarettes often have difficulty quitting with traditional therapies and therefore may be willing to try an alternative therapy, such as psilocybin. Addressing major concerns of this population when designing and promoting the study will be key to successful recruitment and enrollment of a psilocybin-assisted therapy study for PWH who smoke cigarettes. Further development, including designing a study protocol, with particular attention to regulatory issues, any potential ethical risks, and IRB considerations in conducting a future study, is needed. Our study to assess acceptability is an initial step in this process.

## Electronic supplementary material

Below is the link to the electronic supplementary material.

Supplementary Material 1

## Data Availability

Data is provided within the manuscript. The data sets generated and analyzed during this study are available from the corresponding author upon request.

## Abbreviations

HIV: Human immunodeficiency virus
PWH: People with HIV
PWH-S: People with HIV who smoke
CO: Carbon monoxide
RA: Research assistant
IRB: Institutional review board
PI: Principal investigator
