# Supplementary material for: “I’ve learned that I’m open-minded to this possibility”: A qualitative study to evaluate the acceptability of a psilocybin-aided smoking cessation treatment for people with HIV who smoke
Source: Addict Sci Clin Pract. 2025 Jul 21;20:56. doi: 10.1186/s13722-025-00563-0 (PMC12278509; doi:10.1186/s13722-025-00563-0)
Supplement: Supplementary file 1 — Supplementary Material 1 [file 13722_2025_563_MOESM1_ESM.pdf]

Updated: 05/31/2023

Project PACS (Psilocybin-Aided Cessation Study)
Qualitative Interview Agenda and Guide

Agenda

Goals and purposes: The facilitator will lead a semi-structured discussion aimed at gathering qualitative information that will provide information interest in use of psilocybin for the purpose of smoking cessation. The goals of the qualitative interview are to gather insights into the overall interest of using psilocybin for the purpose of smoking cessation and to assess the feasibility of designing a pilot study for psychedelic-facilitated smoking cessation treatment among individuals living with HIV.

Study Design: Each interview will be one-on-one. Interviews will be conducted either in-person or via Zoom.

> For in-person: Set up the room so that chairs face one another. Ensure that the door is closed, and the room is private and confidential.
>
> For Zoom: Set up the Zoom invite through Brown Zoom account. Ensure that the participant is alone, and that the RA is in private space. Record the session once consent has been obtained and enable the audio transcript option.

Staffing: Each interview will require a single facilitator who will ask the questions and operate the recording equipment.

Equipment requirements: computer with internet capability and access to Zoom.

Other materials: Interview guide.

Data collection and preliminary analysis: We anticipate approximately 30 minutes of time per interview.

Agenda: This qualitative interview agenda will serve as the basic guide for the interview. What was learned will be summarized on a section-by-section basis. Data analysis for this portion of the study will be a transcript- and note-based analysis. The facilitators and PI will meet to discuss the content and thematic results of the interviews and together will develop a final report regarding the acceptability of this product and implications for future grant proposals.

Research Identification Numbers: Participants will be identified by their study ID number only in reports and transcripts. Names or pseudonyms used during the interview will be edited out of all printed data and replaced with the study ID number during the transcription process. All questions are intended to guide the facilitator to gather the data that investigators are seeking. Transcription will be done verbatim; all personal identifying information will be de-identified. The facilitator should consider the best way to gather the data and rephrase questions as needed. Additionally, the agenda has been set by the team with the intention of providing what would be a natural flow of conversation. It is not necessary to stick to the exact “flow,” but it is necessary to cover each topic and its subtopics. If the participants lead the discussion along a different path, use it to get the greatest depth of data possible and return to earlier sections as necessary.

Updated: 05/31/2023

Interview Guide

Participant Study ID: _______

Interview Date: _____/_____/_____

Interview Time: ____:____ a.m./p.m. – ____:____a.m./p.m.

Start Recording

A. General Perceptions

1. Have you had any previous experience with psychedelics (mushrooms)?
    a. How would you describe those experiences?
    b. When did you last use psychedelics?
    c. Which psychedelics have you tried?

2. Would you consider participating in a research study examining the use of psychedelics?
    a. How would you feel about trying it for a medical reason?
    b. What are your thoughts about using it for the purpose of quitting smoking?

B. Health Perceptions

1. Thinking about your knowledge of psychedelics, do you think there are health benefits or risks to using psychedelics?
    a. What do you think the benefits are?
    b. What concerns do you have about using psychedelics?
    c. What concerns do you have, if any, about using psychedelics to quit smoking?

2. What would you need to be comfortable participating in a psilocybin micro dosing session?

    Probe: There is some evidence that psilocybin treatment can be helpful for depressive symptoms and PTSD. We know that people with depressive symptoms have more difficulty quitting smoking. Would that make psilocybin treatment attractive to you, if we were treating smoking dependence and depressive symptoms or PTSD?

C. Wrap Up/Conclusion

1. We talked a lot today about psychedelic-facilitated smoking cessation treatment.
2. Is there anything else that you would like to add?
3. What do you think are the main things we have learned from this discussion today?

Thank you for your time and your help in making this project successful.

Additional – Interview Observations:
